# MENTAL HEALTH AND WELL-BEING OF OLDER CARERS DURING THE COVID-19 PANDEMIC: EVIDENCE FROM ENGLAND

**DOI:** 10.1093/geroni/igac059.2104

**Published:** 2022-12-20

**Authors:** Giorgio Di Gessa, Debora Price

**Affiliations:** UCL, London, England, United Kingdom; The University of Manchester, Manchester, England, United Kingdom

## Abstract

Older people caring at home or in the community play a vital role in supporting population health and wellbeing and in protecting health and care systems, often at cost to their own health. Yet there has been very little research or policy attention given to this group of carers during the pandemic. Exploiting longitudinal data from Wave 9 (2018/19) and the first two COVID-19 sub-studies (June/July 2020; November/December 2020) of the English Longitudinal Study of Ageing, we use logistic and linear regression models to investigate associations between changes in provision of informal care and mental health during the pandemic, controlling for socio-demographic characteristics, pre-pandemic physical and mental health, and social isolation measures. During the first months of the pandemic, about a quarter of older people provided informal care (with ~10% caring for members living in the same household). Those caring in the household experience worse mental health during the pandemic. Even controlling for prior characteristics and lack of social interactions, those caring for family members in the household had higher odds of reporting elevated depressive symptoms (OR=1.67, 95%CI=1.07;2.62), poor self-rated health (OR=1.73, 95%CI=1.09;2.73), anxiety (OR=2.21, 95%CI=1.20;4.06) as well as lower quality of life (B=-0.85, 95%CI=-1.66;-0.05) and life satisfaction (B=-0.43; 95%CI=-0.78;-0.09) than those who were caring for friends and family outside the household. As we aim to build back society and restore the wellbeing of our populations, policies and services should be better directed to support those people who during the pandemic struggled to cope while caring for their family members.

